# High-fat diet induced obesity primes inflammation in adipose tissue prior to liver in C57BL/6j mice

**DOI:** 10.18632/aging.100738

**Published:** 2015-04-23

**Authors:** Roel A van der Heijden, Fareeba Sheedfar, Martine C Morrison, Pascal PH Hommelberg, Danny Kor, Niels J Kloosterhuis, Nanda Gruben, Sameh A Youssef, Alain de Bruin, Marten H Hofker, Robert Kleemann, Debby PY Koonen, Peter Heeringa

**Affiliations:** ^1^ University of Groningen, University Medical Center Groningen, Department of Pathology and Medical Biology, Section Medical Biology, Groningen, The Netherlands; ^2^ University of Groningen, University Medical Center Groningen, Department of Pediatrics, Section Molecular Genetics, Groningen, The Netherlands; ^3^ Department of Metabolic Health Research, Netherlands Organization for Applied Scientific Research (TNO), Leiden, The Netherlands; ^4^ Dutch Molecular Pathology Center, Department of Pathobiology, Faculty of Veterinary Medicine, Utrecht University, Utrecht, The Netherlands; ^5^ Radboud University Medical Center, Department of Physiology, Nijmegen, The Netherlands

**Keywords:** obesity, metabolic syndrome, insulin resistance, inflammation, adipose tissue, NASH, liver

## Abstract

Metabolic inflammation in adipose tissue and the liver is frequently observed as a result of diet-induced obesity in human and rodent studies. Although the adipose tissue and the liver are both prone to become chronically inflamed with prolonged obesity, their individual contribution to the development of metabolic inflammation remains speculative. Thus, we aimed to elucidate the sequence of inflammatory events in adipose and hepatic tissues to determine their contribution to the development of metabolic inflammation and insulin resistance (IR) in diet-induced obesity. To confirm our hypothesis that adipose tissue (AT) inflammation is initiated prior to hepatic inflammation, C57BL/6J male mice were fed a low-fat diet (LFD; 10% kcal fat) or high-fat diet (HFD; 45% kcal fat) for either 24, 40 or 52 weeks. Lipid accumulation and inflammation was measured in AT and liver. Glucose tolerance was assessed and plasma levels of glucose, insulin, leptin and adiponectin were measured at various time points throughout the study. With HFD, C57BL/6j mice developed a progressive obese phenotype, accompanied by IR at 24 and 40 weeks of HFD, but IR was attenuated after 52 weeks of HFD. AT inflammation was present after 24 weeks of HFD, as indicated by the increased presence of crown-like structures and up-regulation of pro-inflammatory genes *Tnf, Il1β, Mcp1* and *F4/80*. As hepatic inflammation was not detected until 40 weeks of HFD, we show that AT inflammation is established prior to the development of hepatic inflammation. Thus, AT inflammation is likely to have a greater contribution to the development of IR compared to hepatic inflammation.

## INTRODUCTION

Obesity and associated metabolic disorders like insulin resistance (IR), type 2 diabetes (T2D) and non-alcoholic fatty liver disease (NAFLD) are marked by a state of inflammation initiated by nutrient overload [1]. Metabolic inflammation in obesity, in contrast to the classical immune response to injury or infection, is modest and without apparent resolution over time [1]. Although it is well established that metabolic inflammation disrupts cellular metabolism and impairs insulin signalling in metabolically active tissues, the underlying mechanisms are as yet ill-defined [2].

Adipose tissue (AT) is an important site of inflammatory events in obesity. It contains various cell types that all contribute to the inflammatory response during obesity. In addition to regulating fat mass and nutrient homeostasis, adipocytes mediate the inflammatory response through the secretion of adipokines, cytokines, and chemokines that enhance the recruitment of immune cells, especially macrophages, to the AT [3]. These AT macrophages (ATMs) are a major source of pro-inflammatory cytokines and chemokines and once activated can propagate the inflammatory state and interfere with insulin sensitivity in insulin target cells [4]. Although, prolonged nutrient overload results in metabolic inflammation associated with adipocyte expansion and dysfunction, metabolic inflammation in obesity is not restricted to the AT; the liver is also a strong contributor to the development of this type of inflammation [5–7]. Indeed, the production of pro-inflammatory cytokines secreted by resident macrophages in the liver is linked to disruption of hepatic insulin signalling [8]. Consistent with this, enhanced expression of inflammatory genes in the liver following high-fat diet (HFD) feeding is associated with reduced insulin sensitivity in mice [9,10]. Thus, metabolic inflammation in both organs is associated with the development of IR. However, what tissue provides the main contribution to the development of IR remains unknown.

Few studies have addressed the temporal relationship between AT and liver inflammation in the etiology of IR. Nevertheless, it is generally accepted that AT is an important initiator of the inflammatory response to obesity [4,11], engaging in crosstalk with the liver to affect liver metabolism and IR [12,13]. On the contrary, others have shown that this crosstalk in the development of HFD-induced metabolic derangements is bidirectional [14,15]. Furthermore, hepato-specific targeting of endoplasmatic reticulum stress was associated with decreased obesity and AT inflammation in *ob/ob* mice [14], underscoring the existence of crosstalk between the liver and the AT in obesity. The sequence of inflammatory processes in adipose and hepatic tissues in respect to the development of IR thus represents a topic of debate.

This study aims to elucidate the initiation and sequence of inflammatory events in the adipose and hepatic tissues in obesity, and to determine the contribution of these individual inflammatory processes to the overall development of metabolic inflammation and IR. We hypothesize that liver inflammation comes secondary to adipose tissue inflammation and is of lesser importance to the development of metabolic inflammation and IR in diet-induced obesity.

## RESULTS

### Body weight gain stabilizes after prolonged HFD-feeding in mice

**Figure 1:**
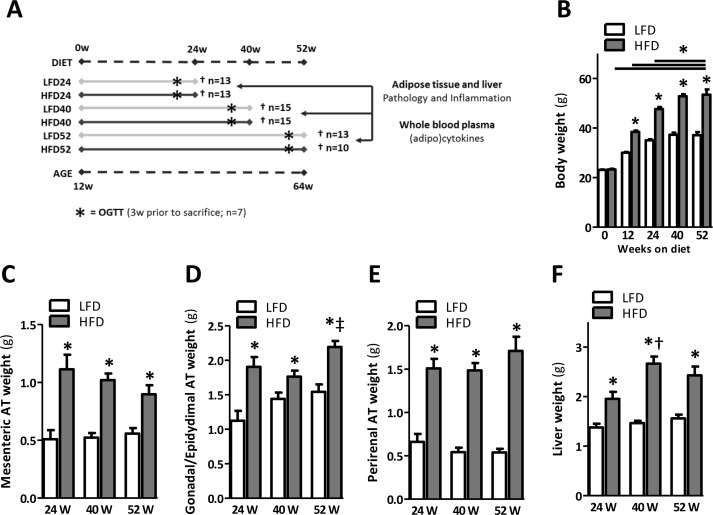
Prolonged HFD-feeding leads to obesity and organ adiposity (**A**) Experimental design. (**B**) Body weights of mice fed a LFD and HFD shown at the start and at 12, 24, 40 and 52 weeks after a HFD. (**C**) Mesenteric, (**D**) gonadal/epidydimal and (**E**) perirenal fat depots weights. (**F**) Liver weight measured upon sacrifice at 24, 40 and 52 weeks. Values shown are means ± SEM (n = 10-15 mice/group). Significance level set at p<0.05. *=significant from LFD at same time point, †=significant from same diet 24w, ‡=significant from same diet 40w.

Body weight and AT mass were measured in mice fed a LFD and HFD for 24, 40 and 52 weeks, at the age of 36, 52 and 64 weeks respectively (Fig. 1A) to assess the development of obesity over time. As expected, we observed a rapid increase in body weight in mice fed a HFD, which was significant from 12 weeks following a diet onwards (Fig. 1B). Body weight gradually increased between mice fed a HFD for 24 weeks and 40 weeks (Fig. 1B; p<0.001) and remained at the same level in mice fed a HFD for 52 weeks compared to HFD for 40 weeks (Fig. 1B). In addition, weights of the mesenteric, gonadal/epidydimal and perirenal fat depots were significantly increased in mice fed a HFD for 24, 40 and 52 weeks as compared to their age-matched LFD controls (Fig. 1C-E). We observed no difference in all three fat depots between mice fed a HFD for 24, 40 or 52 weeks (Fig. 1C-E), suggesting a steady degree of adiposity following its initial establishment. Liver weight was significantly higher in mice fed a HFD for 24, 40 and 52 weeks as compared to LFD controls (Fig. 1F).

### Improved insulin sensitivity upon prolongation of HFD-feeding in mice

**Figure 2:**
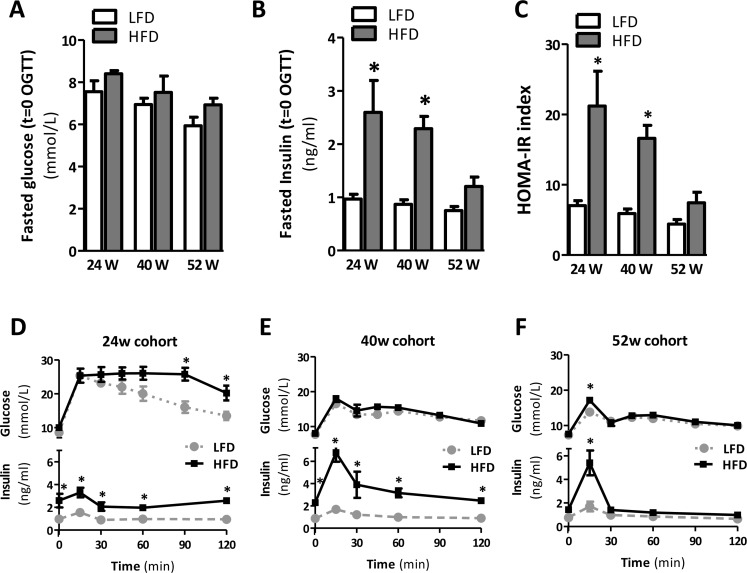
Prolonged HFD is associated with IR after 24 and 40 weeks but not after 52 weeks of HFD (**A**) Fasted plasma glucose and (**B**) insulin concentrations measured in blood collected prior to oral glucose tolerance test (OGTT). (**C**) Homeostatic model assessment of IR (HOMA-IR) used as a surrogate marker of IR. (**D-F**) Blood glucose (top) and plasma insulin (bottom) levels during OGTT after 24, 40 and 52 weeks of LFD and HFD-feeding. Values shown are means ± SEM (n=7 mice/group). Significance level set at p<0.05. *=significant from LFD at same time point, †=significant from same diet 24w, ‡=significant from same diet 40w.

Since obesity is associated with IR, we next monitored insulin sensitivity in mice fed a HFD for 24, 40, and 52 weeks. In mice fed a HFD at all points in time the fasted glucose levels were not affected (Fig. 2A), but fasted insulin levels in mice fed a HFD for 24 and 40 weeks were significantly elevated (Fig. 2B), suggesting a reduction in insulin sensitivity in these mice. Furthermore, we performed an oral glucose tolerance test (OGTT) and measured glucose and insulin levels simultaneously during the OGTT. At 24 weeks of HFD feeding, glucose tolerance was negatively affected, as evidenced by elevated glucose levels during the OGTT (Fig. 2D; p<0.01 at t=90 and p<0.05 at t=120, Suppl. 1A; p<0.05) when compared to age-matched LFD controls. Furthermore, insulin levels were significantly increased in 24-week HFD fed mice compared to age-matched LFD controls (Fig. 2D, Suppl. 1B), confirming the existence of IR at this point in time. Whole-body glucose tolerance was also significantly impaired in mice fed a HFD for 40 weeks, as mice displayed elevated insulin levels during the OGTT (Fig. 2E, Suppl. 1B), whereas blood glucose levels remained stable (Fig. 2E, Suppl. 1A). In contrast, we observed no signs of apparent glucose intolerance in mice fed a HFD for 52 weeks, as glucose levels did not differ (Fig. 2F, Suppl. 1A) and plasma insulin was significantly increased only after 15 min following gavage (Fig. 2F, Suppl. 1B). In support of the glucose tolerance data, homeostasis model assessment of IR (HOMA-IR) showed values to be significantly higher in the 24- and 40-week HFD-fed mice as compared to their age-matched LFD controls (Fig. 2C); this suggests a state of IR in mice fed a HFD for 24 and 40 weeks. HOMA-IR was not increased in mice fed a HFD for 52 weeks, confirming the absence of IR in these mice (Fig. 2C).

### Inflammation in AT is apparent at 24 weeks of HFD feeding

**Figure 3:**
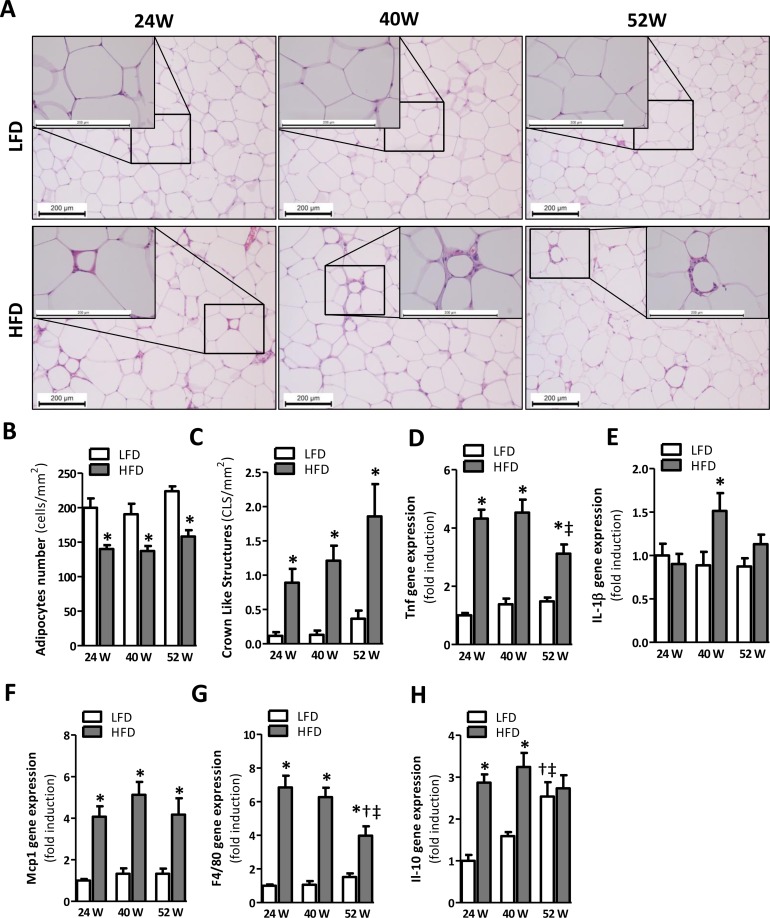
Prolonged HFD-feeding leads to AT inflammation in mice after 24 weeks **(A)** Representative pictures from H&E-stained AT sections of LFD and HFD mice after 24 (left), 40 (middle) and 52 (right) weeks of diet with crown-like structures (insets). Histologically quantified number of **(B)** adipocytes and **(C)** crown-like structures per mm^2^ (n=10-15). **(D-H)** mRNA expression levels of tumor necrosis factor (*Tnf*), interleukin-1β (*Il1β*), monocyte chemotactic protein-1 (*Mcp1*), macrophage marker (*F4/80*) and interleukin-10 (*Il-10*) in the AT. All mRNA expression data were normalized to the LFD24 group and expressed as mean ± SEM (n=7-8). Significance level set at p<0.05. *=significant from LFD at same time point, †=significant from same diet 24w, ‡=significant from same diet 40w.

Hematoxylin and Eosin (H&E) staining of gonadal (epididymal) fat tissues (Fig. 3A) suggested an increased adipocyte size in mice fed the HFD. The number of cells per mm^2^ was significantly reduced by 24, 40, and 52 weeks of HFD-feeding, confirming an increased adipocyte size when compared to LFD age-matched controls (Fig. 3B). Furthermore, crown-like structures (CLS), representing an accumulation of macrophages around dead adipocytes, were apparent in AT samples of mice fed a HFD for 24, 40 and 52 weeks (Fig. 3A, insets). Scoring of CLS confirmed a significant difference to exist between LFD and HFD mice for every time point (Fig. 3C). To confirm the presence of AT inflammation, we next assessed the expression of various inflammatory genes. Indeed, the gene expression levels of the pro-inflammatory cytokine *Tnf* (Fig. 3D) and the genes encoding for proteins involved in macrophage infiltration, *Mcp1* and *F4/80* (Fig. 3E-G), were significantly elevated in the adipose tissue of 24-, 40- and 52-weeks HFD mice compared to LFD mice. *Il1ß* was only significantly elevated in HFD mice at the 40-weeks time point (Fig. 3E). Besides these pro-inflammatory genes, the anti-inflammatory gene *Il-10* was significantly elevated in the AT of HFD mice at 24 and 40 weeks (Fig. 3H). Furthermore, we observed a decrease in *Tnf* and *F4/80* expression in 52-weeks HFD fed mice compared to mice fed a HFD for 40 weeks (Fig. 3D, *Tnf*; p<0.05 and Fig. 3G, *F4/80*; p<0.05), whereas *Il-10* expression increased over time in LFD mice (Fig. 3H, 52-weeks LFD *vs* 24-weeks LFD: p<0.001; 52-weeks LFD *vs* 40-weeks LFD: p<0.05).

### Hepatic inflammation gradually develops over time in mice fed a HFD

**Figure 4:**
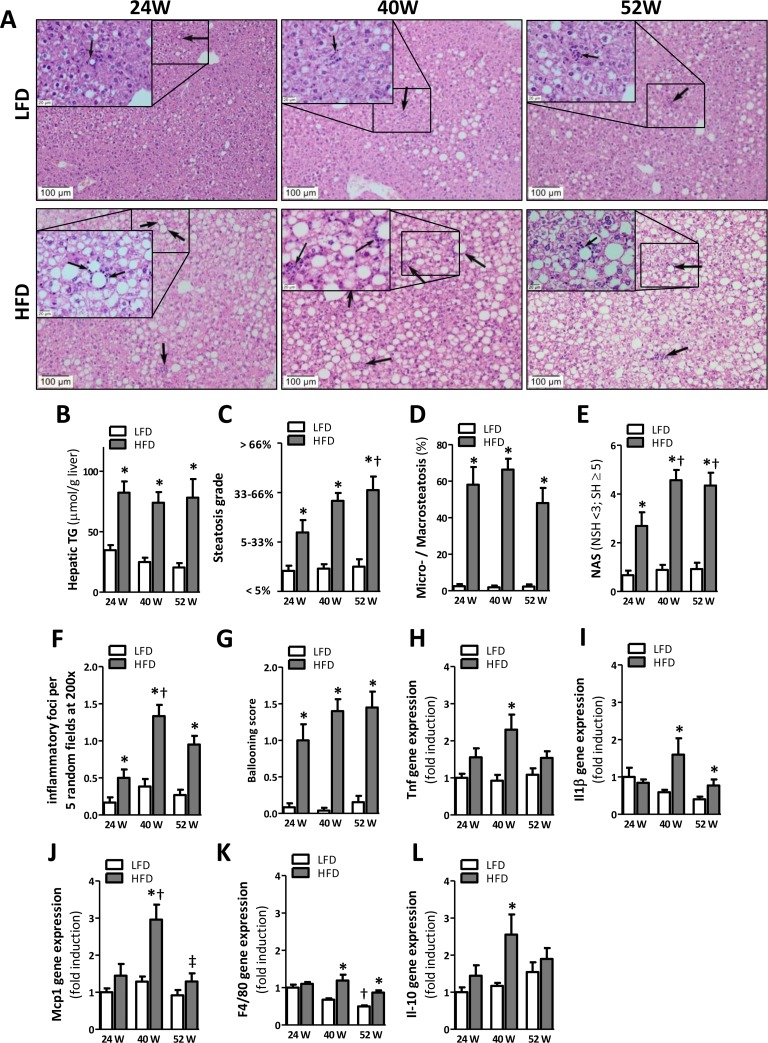
HFD-feeding leads to hepatic steatosis after 24 weeks but inflammation becomes apparent after 40 weeks (**A**) Representative pictures from H&E-stained liver sections of LFD and HFD mice after 24 (left), 40 (middle) and 52 (right) weeks of diet with inflammatory foci (arrows; insets). (**B**) Quantification of hepatic triglycerides. (**C**) Determination of steatosis grade (0=0-5%; 1=5-33%; 2=33-66%; 3=66-100% coverage), and (**D**) percentage of microvesicular over macrovesicular steatosis. (**E**) Determination of NAFLD Activity Score (NAS; sum of steatosis + lobular inflammation + ballooning), score >5 represents steatohepatitis (SH) and pathological scores <3 non-steatohepatitis (NSH). (**F**) Quantification of inflammatory foci per 5 random fields under 200x magnification and (**G**) determination of ballooning score. (**H-L**) mRNA expression levels of tumor necrosis factor (*Tnf*), monocyte chemotactic protein-1 (*Mcp1*), macrophage marker (*F4/80*), interleukin-1β (*Il1β*), and interleukin-10 (*Il-10*) in the AT. All mRNA expression data were normalized to the LFD24 group and expressed as mean ±SEM (n=7-8). Significance level set at p<0.05. *=significant from LFD at same time point, †=significant from same diet 24w, ‡=significant from same diet 40w.

H&E staining of liver slides (Fig. 4A) indicated the presence of HFD-induced hepatic steatosis from 24 weeks onwards, which was confirmed by a significant elevation in the amount of neutral lipids in the liver (TG; Fig. 4B). Histopathological examination of liver sections showed an increase in steatosis grade at 52 weeks as compared to 24 weeks of HFD-feeding (Fig. 4C; p<0.05), suggesting a progression in the accumulation of hepatic lipids over time. Although prolonged HFD-feeding significantly increased the percentage of microvesicular steatosis over macro-vesicular steatosis, it did not differ over time (Fig. 4D).

Furthermore, we observed a significant increase in the NAFLD activity score (NAS) (Fig. 4E), the number of inflammatory foci (Fig. 4F) and hepatocellular ballooning (Fig. 4G) for all HFD groups as compared to LFD groups. In line with a progression in steatosis grade, we also observed an increase in both the NAS score (Fig. 4E; p<0.05) and the number of inflammatory foci (Fig. 4F; p<0.001) in mice fed a HFD for 40 weeks as compared to the 24-weeks HFD group. To further investigate inflammation we measured the expression of pro- and anti-inflammatory cytokines (*Tnf, Il1ß, Il-10*) and macrophage markers (*Mcp1, F4/80*) in the liver. *Tnf, Il1ß, Il-10, Mcp1, and F4/80* expression was significantly upregulated at 40 weeks of HFD-feeding but not 24 weeks of HFD-feeding compared to mice fed the LFD. Furthermore, no difference was observed in expression level of *Tnf*, *Mcp1* and *Il-10* at 52 weeks of HFD-feeding, whereas *Il1ß* and *F4/80* were still significantly elevated at this time point (Fig. 4H-L). In addition, *Mcp1* expression was significantly reduced in HFD52 mice compared to HFD40 mice (Fig 4I). We also observed an age-related decline in *F4/80* expression as represented by a significant reduction in *F4/80* in LFD52 mice compared to LFD24 mice (Fig. 4K).

### Plasma adipokine and cytokine levels during prolonged HFD-feeding in mice

**Figure 5:**
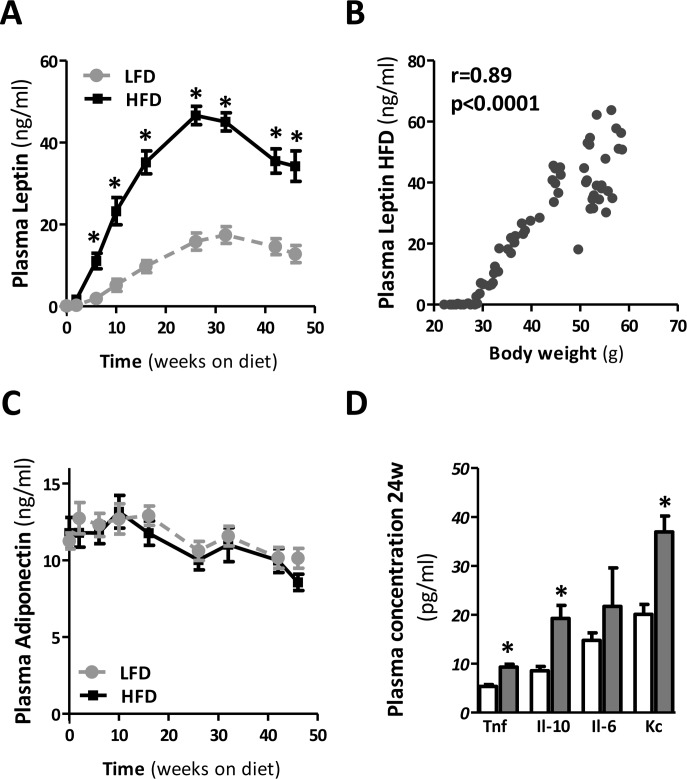
Plasma adipokine and cytokine levels during prolonged HFD in mice (**A**) Serial plasma leptin and (**B**) its correlation with body weight, and (**C**) serial plasma adiponectin throughout the dietary intervention. (**D**) Plasma cytokine levels in mice fed a LFD and HFD for 24 weeks. (n=10-15 mice/group). Significance level set at p<0.05. *=significant from LFD at same time point.

Both leptin and adiponectin are associated with obesity, IR and T2D. To investigate the extent to which prolonged HFD-feeding mediates the secretion of leptin and adiponectin, we measured the plasma levels of these adipokines throughout the dietary period. As expected, leptin was significantly increased in mice fed a HFD compared to LFD, reaching a maximum around 24 weeks (Fig. 5A). Plasma leptin concentrations exhibited very strong correlation with body weight (Fig. 5B). Serial adiponectin levels did not change in time or as a result of HFD-feeding (Fig. 5C). After 24 weeks of HFD, the circulating pro-inflammatory mediators, TNF, IL-6, and mKC, as well as the anti-inflammatory mediator IL-10 were up-regulated (Fig. 5D), confirming immunological activation in HFD-fed mice.

## DISCUSSION

To date, the detrimental role of metabolic inflammation in the etiology of IR has become irrefutable [10]. Despite the fact that inflammation in AT and liver are both associated with IR, their individual contribution to the development of metabolic inflammation remains elusive. Here, we have explored the temporal relationship between AT and liver inflammation in the development of metabolic inflammation. We show that chronic metabolic inflammation is apparent in the visceral AT depot at 24 weeks of HFD-feeding (Fig. 3), whilst overt hepatic inflammation does not occur until 40 weeks of HFD-feeding (Fig. 4). This implies that AT inflammation precedes hepatic inflammation in mice fed an obesogenic diet. Furthermore, as IR was established before the development of clear hepatic inflammation and did not further progress, our data also suggest that obesity-associated IR is more likely to be associated with AT inflammation rather than hepatic inflammation.

In agreement with previous reports demonstrating that HFD-feeding favours the development of obesity [10,16], our data show that body weight gain increased rapidly within the initial 12-24 weeks of HFD-feeding (Fig. 1B). Prolonged HFD-feeding in mice led to a further, yet marginal, increase in body weight up to a maximum at about 40 weeks of HFD-feeding. Weight gain then levelled off and weight remained high until the end of the study. Although we used male mice in our study, previous studies observed a similar growth pattern in female mice fed a HFD-diet (60% kcal fat) for up to 12 months [16]. In the female mice the increase in body weight was greater during the first 12 weeks and slower during subsequent weeks of HFD-feeding. Correspond-ing to the rate of body weight gain in mice fed a HFD, plasma leptin levels were significantly increased compar-ed with mice fed a LFD (Fig. 5). Indeed, as adipocytes expand due to enhanced triglyce-ride storage, leptin secretion increases proportionately [17]. However, leptin levels significantly declined with prolonged HFD-feeding (±30 weeks of HFD-feeding) although body weight remained high (Fig. 5A, B). Leptin levels also declined in mice fed the LFD, suggesting an age-related decline in circulating leptin. This has previously been observed in rodents [18] and also in the human population leptin levels have been found to decline with age [19].

Although AT is an important initiator of the inflammatory response to obesity [4], few studies have addressed the temporal relationship between AT and the liver in the development of metabolic inflammation. Here we show that 24 weeks of diet-induced obesity indeed led to an increase in AT inflammation. In contrast to *Tnf, Mcp1* and *F4/80, Il1β* was not elevated at the 24-week time point (Fig. 3D). However, we cannot fully exclude that Il1β was not expressed at this time point, since cytokine expression was not measured in the stromal vascular fraction but in AT lysates only, which may have led to an underrepresentation of the results. Of note, gene expression of the anti-inflammatory cytokine *Il-10* was persistently elevated in HFD mice as well. This is in agreement with previous studies in mice [20] and humans [21] and likely represents a protective response of the body to counteract pro-inflammatory events.

Other longitudinal studies investigating the development of adipose and liver inflammation found that both organs exhibit a very similar response to HFD feeding at the level of transcriptional master regulators [22]. However, the response at the level of genes controlled by these regulators was tissue-specific and, importantly, the authors observed a delayed response in the liver when compared to the AT, which is consistent with our findings. What causes this delayed inflammatory response remains to be elucidated. However, it has been shown that systemically administered cytokines can exert pro-inflammatory effects on the liver. Especially TNF and leptin are known to play a role in the pathogenesis of NAFLD [23,24]. Here, we show that AT inflammation is associated with the enhanced secretion of TNF and leptin to the circulation after 24w of HFD (Fig. 5). Since the visceral AT depot directly drains into the portal circulation, this may support the notion of crosstalk between dysfunctional AT and the liver. Thus, our results indicate that metabolic inflammation in obesity is initiated in the AT and in time progresses to the liver. Therefore, our data suggest that the liver does not play a role in the initial development of metabolic inflammation in mice but may serve as a contributor to metabolic inflammation following its establishment.

Aging is associated with increased visceral adiposity [25,26], AT inflammation and a systemic increase in pro-inflammatory cytokines [27], thus closely resembling the pathophysiology in obesity. Given that this inflamm-aging is linked with various age-associated disorders [28], targeting inflammatory pathways could prevent or alleviate the pathological consequences observed in the course of aging, obesity or both in parallel. Over the last decade mTOR inhibitors, especially rapamycin, have shown promising effects on extending life- and health span in animal models of aging [29,30] and obesity [31], effects that, at least for obesity, may be attributed to rapamycin's anti-inflammatory properties in the AT [32]. Whether this may be applied to the human setting to increase health span in the growing aging population remains unknown.

Our data also show that IR was induced in mice fed a HFD for 24 weeks compared with LFD mice. Since at this point AT inflammation was present, in the absence of clear hepatic inflammation, this may suggest AT inflammation is more likely to contribute to obesity-associated IR. This observation accords with our previous reports regarding the dissociation of hepatic inflammation and IR and suggests a greater contribution of adipose inflammation to development of IR than hepatic inflammation [33,34]. Our study design, however, does not allow for assessing whether AT inflammation triggers systemic IR or vice versa since at our earliest time point (24 weeks HFD-feeding) both IR and AT inflammation were observed. It has, however, been shown previously that AT inflammation is necessary for long-term but not short-term HFD-induced IR, which is more likely related to acute tissue lipid overload [10].

Nevertheless, our data also show that prolonged HFD feeding is associated with improved insulin sensitivity over time, as we did not observe signs of glucose intolerance in mice fed a HFD for 52 weeks (Fig. 2, Suppl. 1). This increase in insulin sensitivity was already observed in mice fed a HFD for 40 weeks compared to 24 weeks. Although the observed alleviation of IR clearly warrants further study it is known that insulin responses and insulin levels decline with age in the human population [35]. This could reflect beta cell failure in aging, or might be due to alterations in gastric emptying, delayed nutrient absorption, or enhanced insulin sensitivity in older age [36]. Furthermore, an age-related decline in renal function may also affect glucose metabolism and impair insulin secretion [36,37]. Whether any of these factors can explain the amelioration of insulin sensitivity in our study remains to be investigated. In addition, the high serum leptin levels as observed in mice fed the HFD may have resulted in inhibition of insulin release from the pancreas [38]. Furthermore, the apparent improvement in insulin sensitivity in our study may also be related to the attenuated inflammatory phenotype of the AT observed with aging. Indeed, AT of mice fed a 52-week diet of HFD had a reduced expression of the inflammatory genes *Tnf* and *F4/80* in comparison with mice fed a HFD for 40 weeks. Although this could reflect the reciprocal relationship between inflammation and IR, it should be noted that the reduced expression of inflammatory genes was not paralleled by a reduced number of crown-like structures in the AT (Fig 3C). A similar trend was observed for the liver, in which *Mcp1* expression was significantly reduced in mice fed a HFD for 52 weeks compared with mice at 40 weeks. This may seem paradoxical at first sight, however, this finding may be explained at the macrophage's functional level where it has been reported that with aging macrophages obtain a reduced capacity to become activated into a pro-inflammatory state by chemokines and cytokines [39], thus allowing a similar amount of macrophages to be associated with a reduced cytokine expression with age. Nevertheless, the influx of macrophages to the AT is less impressive with aging and it has been suggested that (pre-) adipocytes, rather than macrophages, are the major source of inflammation in aging [40]. Although we cannot discriminate between the different cell types involved in AT and liver inflammation, aging by itself did not result in upregulation of pro-inflammatory genes and cytokines in LFD52 mice. This is in discrepancy with the study by Wu *et al.* and may be explained by the longer duration (22-24 months) and different strain of mice (C57BL/6JNIA) used in their study. Nevertheless, *Il-10* was significantly increased in time suggesting some degree of age-induced immunological activation in our study.

Taken together, we provide evidence that obesity-induced metabolic inflammation in the AT precedes inflammation in the liver, suggesting that the liver does not play a role in the initial development of metabolic inflammation. Furthermore, we show that hepatic inflammation is of lesser importance to the development of insulin resistance compared to AT inflammation. Therefore, targeting AT inflammation may help to control metabolic inflammation in obesity and aging in mice by both delaying its initiation and subsequent progression to other organs such as the liver. Further research is warranted for successful extrapolation of the research results to human clinical conditions.

## MATERIALS AND METHODS

### Animals and diet intervention

All procedures were performed with approval of the University of Groningen Ethics Committee for Animal Experiments (experiment registered as 6141B), which adheres to the principles and guidelines established by the European Convention for the Protection of Laboratory Animals. Experiments were carried out on male C57BL/6j mice (Charles River, JAX laboratories, France), individually housed in a temperature-controlled room under a 12 h light-dark cycle with *ad libitum* access to water and food, unless stated otherwise. After arrival at our laboratories the 6-week old C57BL/6j male mice received a low-fat diet (LFD) containing 10% fat from lard (D12450HY, Research Diets Inc., NJ, USA) for 6 weeks until the start of the experiment, at which time the animals were 12 weeks old. After this run-in period, randomly divided groups of animals were either kept on the LFD or switched to a high-fat diet (HFD) containing 45% fat from lard (D12451, Research Diets Inc.). Experiments were performed on mice after 24, 40 and 52 weeks of HFD-feeding unless stated otherwise. To ensure that observations took place in a state of chronic obesity, animals exhibiting a weight loss >15% at sacrifice, compared with their peak body weight were excluded from the analyses. Six mice were excluded from the study due to the development of dermatitis, that is n=2 in the HFD24 group, n=3 in the HFD52 group, and n=1 in the LFD52 cohort. Final group sizes were n=13 for LFD24, n=13 for HFD24, n=15 for LFD40, n=15 for HFD40, n=13 for LFD52 and n=10 for HFD52.

### Oral glucose tolerance test

Three weeks prior to sacrifice subsets of mice (n=7 per group) were fasted for 6 hours and received a glucose bolus (2 g/kg; 40% glucose solution (w/v) by oral gavage. Average gavage volume was 180±13 and 250±24 μl for LFD- and HFD-mice, respectively. Glucose levels were measured with the OneTouch Ultra glucometer (Lifescan Benelux, Beerse, Belgium) before and 15, 30, 45, 60, 90, and 120 minutes after the gavage by puncturing the saphenous vein of the left hind limb. Blood samples collected prior to glucose administration and at 15, 30, 60 and 120 minutes were used to simultaneously assess plasma insulin levels. HOMA-index for systemic IR was calculated as described elsewhere [41].

### Analysis of plasma parameters

Plasma insulin was determined by ELISA (Alpco ultrasensitive, Alpco, Tilburg, The Netherlands). Plasma leptin and adiponectin were determined by ELISA (R&D systems, Abingdon, UK). Plasma levels of interleukin IL-1β, IL-2, IL-5, IL-6, IL-10, IL-12 and mKC (CXCL-1), p70, TNF and IFN-γ were measured using Meso Scale Discovery (Gaithersburg, USA) 10-plex multispot Mouse cytokine assay for plasma, according to the manufacturer's instructions. Intra- and inter-assay coefficients of variation were, respectively, IL-10 (15.8 and 31.2%), IL-6 (11,5 and 3.3%), mKC (8.3 and 13.7%) and TNF (6.4 and 16.3%). Concentrations of IFN-γ, IL-12, p70, IL-1β, IL-2, IL-5 were below the detection limit of the assay.

### Liver and AT Histology

Three different visceral adipose depots (i.e. surrounding the intestine; mesenteric, the kidney; perirenal, and the gonads; gonadal/epidydymal) were isolated and weighed at sacrifice. Subsections were partially embedded in paraffin and snap frozen and stored at −80°C for further analyses. The frozen gonadal fat depot was crushed in liquid nitrogen for RNA isolation. Livers were collected, trimmed, and trimmed sections were fixed in 10% paraformaldehyde. Paraffin-embedded liver and AT sections (4 μm) were stained with Hematoxylin/Eosin (H&E) for morphological examination. Scoring of hepatic steatosis and steatohepatitis (NAFLD activity score NAS) was performed in an unbiased manner by two board-certified veterinary pathologists (SAY, AdB) using a method described previously [42].

To estimate adipocyte size, the number of adipocytes per mm^2^ was determined on scanned sections by manually counting all the adipocytes in an area of 4-5 mm^2^, using Aperio ImageScope (Leica Biosystems Imaging Inc., CA, USA). Slides were scanned with the NanoZoomer 2.0-HT slide scanner from Hamamatsu (Herrsching am Ammersee, Germany).

### Liver lipids

Total liver lipids were extracted from the liver according to the Bligh and Dyer method [43], as previously explained [44], and measured biochemically. Hepatic triglycerides (TG) were quantified using commercially available kits (Hitachi, Roche, Woerden, The Netherlands).

### Quantitative Real-Time PCR (qRT-PCR)

Total RNA was isolated from the liver and AT using RNeasy Mini plus and RNeasy Lipid Tissue Mini kits (Qiagen, Wetburg, Leusden, The Netherlands), respectively, according to the manufacturer's instructions. RNA integrity was determined by agarose gel electrophoresis. RNA quantity (OD-260) and quality (260/OD-280) were determined using an ND-1,000 spectrophotometer (NanoDrop Technologies, Rockland, DE). Total RNA (1 μg) was converted into cDNA using a Quantitect Reverse Transcription kit (Roche, Mannheim, Germany) according to the manufacturer's instructions. Real time PCR (RT-PCR) was performed using a 7900HT system (Applied Biosystems, Warrington, UK) as previously explained [45] by using Power SYBR Green Master Mix (Roche, Mannheim, Germany). Values were corrected using the housekeeping gene Cyclophillin A (*Ppia*). Primer sequences are available in Supplementary Table 1.

### Statistical analysis

Data are expressed as means ± standard error of the mean (SEM). Data were statistically analysed using GraphPad Prism (version 5.00 for Windows, GraphPad Software, San Diego, CA, USA). Non-parametric Mann-Whitney U tests were performed for comparing LFD and HFD groups within a time point, and Kruskall Wallis ANOVA with Dunn's post hoc test was used to determine differences between time points within the designated diet. Statistical analyses with p-value smaller than 0.05 were considered significant

## SUPPLEMENTARY FIGURE AND TABLE


