# Supplementary material for: High-fat diet induced obesity primes inflammation in adipose tissue prior to liver in C57BL/6j mice
Source: Aging (Albany NY). 2015 Apr 23;7(4):256–67. doi: 10.18632/aging.100738 (PMC4429090; doi:10.18632/aging.100738)
Supplement: Supplementary file 1 [file aging-07-256-s001.pdf]

SUPPLEMENTARY MATERIAL

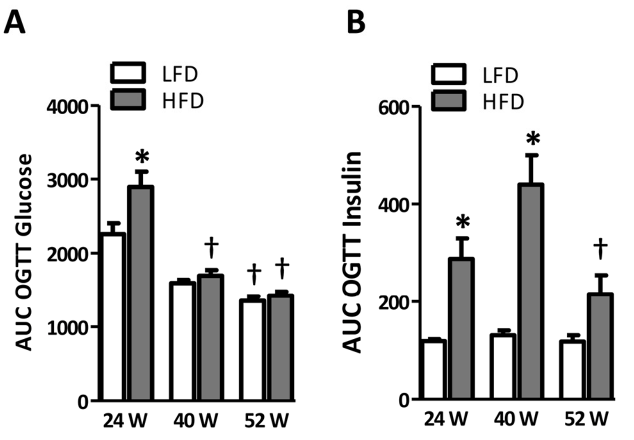

Supplementary Figure 1. Glucose and insulin during OGTT. (**A**) Glucose and (**B**) insulin homeostasis during oral glucose tolerance test (OGTT) quantified by area under curve (AUC) based on data in Fig. 2B-E. Significance level set at $p<0.05$. *=significant from LFD at same time point, †=significant from same diet 24w, ‡=significant from same diet 40w.

Supplementary Table 1. Primer sequences applied for gene expression analyses in AT and liver. Primer sequences for *Tnf, Mcp1, Il-1β, F4/80, Ppia* and *Il-10*

Gene	Forward primer 5'-3'	Reverse primer 5'-3'
F4/80	TGTGTCGTGCTGTTCAGAACC	AGGAATCCCGCAATGATGG
Mcp1	GCTGGAGAGCTACAAGAGGATCA	ACAGACCTCTCTCTTGAGCTTGGT
Il1β	TGCAGCTGGAGAGTGTGG	TGCTTGTGAGGTGCTGATG
Il10	GCTCTTACTGACTGGCATGAG	CGCAGCTCTAGGAGCATGTG
Tnf	GTAGCCCACGTCGTAGCAAAC	AGTTGGTTGTCTTTGAGATCCATG
Ppia	TTCCTCCTTTCACAGAATTATTCCA	CCGCCAGTGCCATTATGG
